# Sample Entropy Combined with the K-Means Clustering Algorithm Reveals Six Functional Networks of the Brain

**DOI:** 10.3390/e21121156

**Published:** 2019-11-26

**Authors:** Yanbing Jia, Huaguang Gu

**Affiliations:** 1 School of Mathematics and Statistics, Henan University of Science and Technology, Luoyang 471000, China; jiayanbing@haust.edu.cn; 2 School of Aerospace Engineering and Applied Mechanics, Tongji University, Shanghai 200092, China

**Keywords:** sample entropy, brain functional networks, complexity, dynamic functional connectivity, static functional connectivity, K-means clustering algorithm

## Abstract

Identifying brain regions contained in brain functional networks and functions of brain functional networks is of great significance in understanding the complexity of the human brain. The 160 regions of interest (ROIs) in the human brain determined by the Dosenbach’s template have been divided into six functional networks with different functions. In the present paper, the complexity of the human brain is characterized by the sample entropy (SampEn) of dynamic functional connectivity (FC) which is obtained by analyzing the resting-state functional magnetic resonance imaging (fMRI) data acquired from healthy participants. The 160 ROIs are clustered into six clusters by applying the K-means clustering algorithm to the SampEn of dynamic FC as well as the static FC which is also obtained by analyzing the resting-state fMRI data. The six clusters obtained from the SampEn of dynamic FC and the static FC show very high overlap and consistency ratios with the six functional networks. Furthermore, for four of six clusters, the overlap ratios corresponding to the SampEn of dynamic FC are larger than that corresponding to the static FC, and for five of six clusters, the consistency ratios corresponding to the SampEn of dynamic FC are larger than that corresponding to the static FC. The results show that the combination of machine learning methods and the FC obtained using the blood oxygenation level-dependent (BOLD) signals can identify the functional networks of the human brain, and nonlinear dynamic characteristics of the FC are more effective than the static characteristics of the FC in identifying brain functional networks and the complexity of the human brain.

## 1. Introduction

The human brain shows complex spatiotemporal behaviors when executing physiological functions. Characterizing dynamics of the complex spatiotemporal behaviors is of great significance in understanding the human brain. Since blood oxygenation level-dependent (BOLD) signals of different brain regions can be measured by the functional magnetic resonance imaging (fMRI) technique at high spatial and temporal resolutions, BOLD signals have been widely used to characterize dynamics of the spatiotemporal behaviors of the human brain [1,2]. For instance, the temporal correlation in BOLD signals of two distinct brain regions is commonly employed to describe the functional connectivity (FC) between them [3]. A positive and strong temporal correlation corresponds to a strong FC, and some brain regions with strong FCs among them constitute a brain functional network [4,5,6]. Alterations of some FCs in a brain functional network are often associated with brain disorder, such as schizophrenia [7], major depression [8], autism [9], Alzheimer’s Disease [10], and attention deficit hyperactivity disorder [11]. For example, Cheng et al. evaluated the FC between different brain regions in subjects with autism and found a key system in the middle temporal gyrus with reduced FC and a key system in the precuneus with reduced FC [12].

In most previous research on FC, only one correlation coefficient is acquired using entire BOLD signals of two distinct brain regions. The one correlation coefficient is called the static FC between the two brain regions. Recently, to understand dynamics of the spatiotemporal behaviors of the human brain more deeply, some researchers acquired a sequence of correlation coefficients by applying the sliding-window approach to BOLD signals of two distinct brain regions [13,14,15,16,17,18,19,20,21,22,23]. These correlation coefficients form a time series which is called the dynamic FC between the two brain regions. The dynamic FC exhibits complex characteristics which are effective in describing properties of the brain functional networks of patients with brain disorder. For instance, in one of our recent studies, complex characteristics of dynamic FC were described by sample entropy (SampEn), and the effects of schizophrenia on such complex characteristics were investigated. It was shown that the visual cortex of the patients with schizophrenia exhibited significantly higher SampEn than that of the healthy controls [24]. As introduced above, both the static FC and the SampEn of dynamic FC are effective in describing properties of the brain functional networks of patients with brain disorder. However, the effectivenesses of the static FC and the dynamic FC have not been compared directly.

Studies on the static FC or the dynamic FC are often carried out by first extracting BOLD signals of different brain regions and then evaluating the static or the dynamic FC between different brain regions for further analysis. Different brain regions are often determined by a brain template, such as the Dosenbach’s template [25]. The Dosenbach’s template includes 160 regions of interest (ROIs) determined by a sequence of meta-analyses of task-based fMRI studies which cover much of the human brain [25]. Furthermore, the 160 ROIs can be separated into six functional networks including the default, the frontal-parietal, the cingulo-opercular, the sensorimotor, the occipital, and the cerebellum networks, which were identified by performing modularity optimization on the average FC matrix across a large cohort of healthy subjects [25]. The six functional networks have been used in predicting brain maturity across development [25,26], parcellating cortical or subcortical regions [27], examining the influence of temporal properties of BOLD signals on FC [28] and so on. For instance, Zhong et al. parcellated the hippocampus based on the FC, and showed that both the left and right hippocampus were divided into three subregions exhibiting different FC profiles with the six functional networks [27]. However, machine learning algorithms have not been used to identify the six functional networks.

The K-means clustering algorithm is one of the unsupervised learning algorithms [29]. Since the K-means clustering algorithm can cluster different observations into different clusters in a simple and easy way, it has been widely used in fMRI studies [30,31,32,33,34,35,36,37,38]. For instance, Fan et al. used the K-means clustering algorithm to parcellate the thalamus based on the static FC and found that the thalamus could be divided into seven symmetric thalamic clusters [36]. Park et al. parcellated the primary and secondary visual cortices (V1 and V2) into several subregions by applying the K-means clustering algorithm to the static FC and found that V1 and V2 could be separated into anterior and posterior subregions [38].

The present study intends to cluster the Dosenbach’s 160 ROIs into six clusters by applying the K-means clustering algorithm to the static FC and the SampEn of dynamic FC, to analyze the overlap and consistency between the six clusters and the six functional networks, and to compare the effectivenesses of the static FC and the dynamic FC. It is shown that applying the K-means clustering algorithm to FC is feasible to identify the six functional networks, and the SampEn of dynamic FC is more effective than the static FC as the six clusters obtained from the SampEn of dynamic FC show higher overlap and consistency ratios with the six functional networks.

This paper is organized as follows. The experiments and methods are presented in Section 2. The cluster results for the static FC and the SampEn of dynamic FC and the comparisons between them are shown in Section 3. The conclusion and discussion are described in Section 4. Some supplementary tables are presented in the appendix.

## 2. Experiments and Methods

### 2.1. Participants

FMRI data for this study were acquired at Olin Neuropsychiatry Research Center and have been made publicly available http://fcon_1000.projects.nitrc.org/indi/abide. The data were acquired from 31 healthy participants (18 males and 13 females) over the age range 18–30 years. This sample was retained after applying criteria for head motion, from a total of 35 healthy participants. Informed consent was obtained from all participants in accordance with Olin Neuropsychiatry Research Center Institutional Review Board oversight.

### 2.2. Data Acquisition and Preprocessing

BOLD signals are extracted from three-dimensional functional images collected on a Siemens 3T MRI scanner with the following parameters: repetition time (TR), 475 ms; echo time, 30 ms; field of view, $240\times 240\;{\mathrm{mm}}^{2}$; slices, 48; slice thickness, 3 mm; flip angle, $60^{\circ }$. During the data collection, all participants were instructed to rest but not fall asleep. For each participant, 947 three-dimensional functional images were collected.

The functional images are preprocessed using SPM8 and DPABI softwares [39,40]. Firstly, the first 4 images are discarded to reduce the negative effects of scanner’s stabilization on the analysis results. Secondly, the images are corrected for time delay in slice acquisition and rigid-body head motion. Thirdly, several confounding factors are regressed out from the images, including 6 head motion parameters and the cerebrospinal, the white matter, and the global brain signals. Fourthly, temporal band-pass filtering (0.01–0.08 Hz) of the images are performed to reduce the negative effects of low-frequency drift and high-frequency physiological noise on the analysis results. Fifthly, the images are spatially normalized to the Montreal Neurological Institute space and are resampled to voxels of size $3\times 3\times 3\;{\mathrm{mm}}^{3}$. Sixthly, the images are smoothed with a Gaussian kernel of 8 mm full-width at half-maximum. Finally, the BOLD signal of each voxel is extracted from the functional images.

### 2.3. The Dosenbach’s Template and the 6 Functional Networks

**Figure 1:**
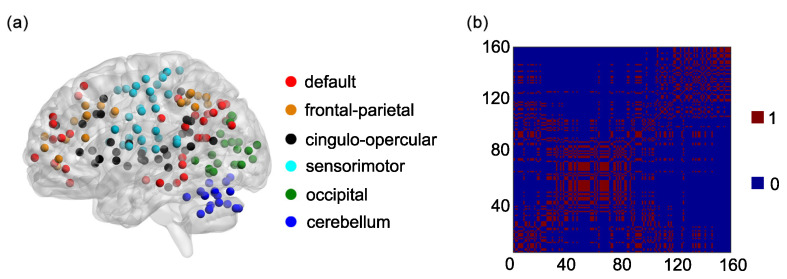
(**a**) One hundred and sixty regions of interest (ROIs) are shown on a surface rendering of the brain. ROIs in different functional networks are shown in different colors. (**b**) The adjacent matrix *A* of 160 ROIs in 6 functional networks.

One hundred and sixty regions of interest (ROIs) are selected based on the Dosenbach’s template [25]. The centroid of each ROI is derived from a sequence of meta-analyses of task-based fMRI studies (Figure 1a). The radius of each ROI equals 5 mm (Figure 1a). The name and the sequential number of each ROI can be found in Table A1 in Appendix A. The 160 ROIs can further be grouped into 6 functional networks, including the default, the frontal-parietal, the cingulo-opercular, the sensorimotor, the occipital, and the cerebellum networks (Figure 1a). The name and the sequential number of each ROI in each functional network can be found in the first and second columns of Table A2, Table A3, Table A4, Table A5, Table A6 and Table A7 in Appendix A.

Based on the 6 functional networks, an adjacent matrix can be generated [36,41,42]. The adjacent matrix is labeled as

(1) $$A=\left[\begin{array}{ccc} a_{1,1} & \cdots & a_{1,160}\\ \vdots & \ddots & \vdots \\ a_{160,1} & \cdots & a_{160,160} \end{array}\right].$$

Each of the elements on the main diagonal of *A* is 1. Other elements of *A* are defined as follows: $a_{i,j}=1$ if the *i*th ROI and the *j*th ROI are contained in the same functional network and $a_{i,j}=0$ otherwise (i,j=1,2,…,160) (Figure 1b).

### 2.4. The Static FC and the Dynamic FC

**Figure 2:**
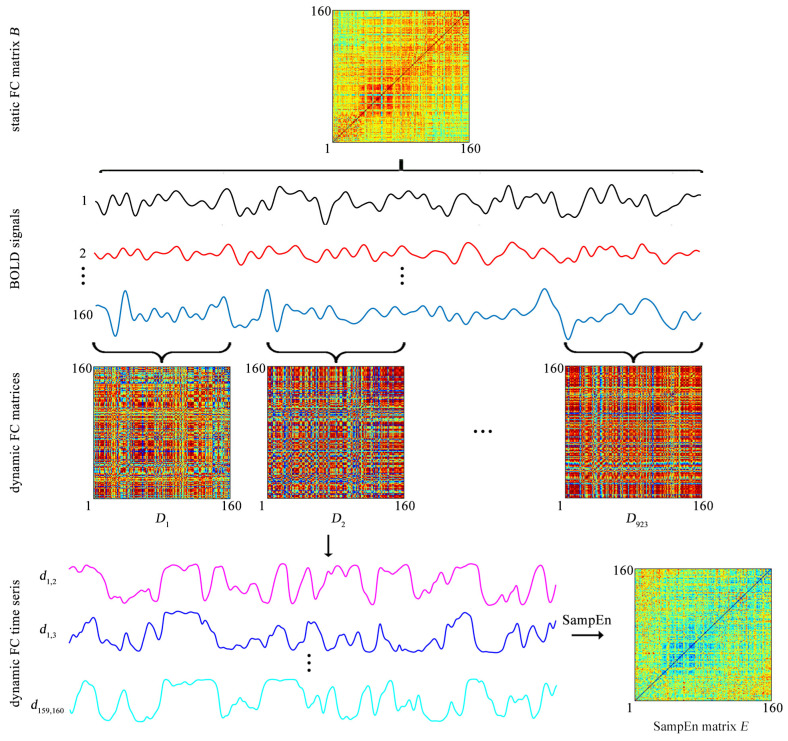
The static functional connectivity (FC) matrix *B* and the SampEn matrix *E* obtained from the BOLD signals of 160 ROIs. The matrices *B* and *E* are used to cluster the 160 ROIs into 6 clusters by the K-means clustering algorithm.

The BOLD signal of each ROI is extracted by averaging the BOLD signals over all voxels in this ROI. Then both the static FC and the dynamic FC are evaluated (Figure 2).

The static FC between each pair of ROIs is assessed by a Pearson correlation coefficient. For each of the 31 participants, after the static FC between each pair of ROIs is evaluated, a static FC matrix of size 160×160 is obtained (Figure 2), which is labeled as

(2) $$B=\left[\begin{array}{ccc} b_{1,1} & \cdots & b_{1,160}\\ \vdots & \ddots & \vdots \\ b_{160,1} & \cdots & b_{160,160} \end{array}\right]=\left[\begin{array}{c} B_{1}\\ \vdots \\ B_{160} \end{array}\right].$$

The *i*th row $B_{i}$ represents the static FC between the *i*th ROI and all the other ROIs (i=1,2,…,160). The matrix *B* is used to cluster the 160 ROIs into 6 clusters.

Dynamic FC is assessed by the sliding-window approach. Specifically, a tapered window is created by convolving a rectangle window (size = 20 TRs = 9.5 s) with a Gaussian curve (standard deviation = 3 TRs) [14,15,23]. The window is used to extract BOLD signals in a step of 1 TR, leading to 923 time windows per subject (Figure 2). For the *k*th time window (k=1,2,…,923), a Pearson correlation coefficient is used to evaluate the FC between each pair of ROIs and thus a FC matrix of size 160×160, which is labeled as

(3) $$D_{k}=\left[\begin{array}{ccc} d_{1,1,k} & \cdots & d_{1,160,k}\\ \vdots & \ddots & \vdots \\ d_{160,1,k} & \cdots & d_{160,160,k} \end{array}\right],$$

which is obtained for each subject (Figure 2). As *k* increases from 1 to 923, $d_{i,j,k}$ forms a time series (i,j=1,2,…,160), which represents the temporal evolution of the FC between the *i*th and *j*th ROIs and is named as the dynamic FC (Figure 2). Since previous studies showed that the window of size 20 TRs captures more transient patterns in dynamic FC [23], the window size is fixed at 20 TRs throughout the study.

### 2.5. SampEn of a Dynamic FC Time Series

For each dynamic FC time series, $d_{i,j}\left(i,j=1,2,\ldots ,160,i\neq j\right),$ the SampEn is calculated. For convenience, time series $d_{i,j}$ is denoted by $x=\left(x_{1},x_{2},\ldots ,x_{N}\right)\left(N=923\right).$ SampEn of x is computed as follows [24,43,44,45,46].

Firstly, constructing embedding vectors $v_{i}=\left(x_{i},x_{i+1},\ldots ,x_{i+m-1}\right),$ in which *m* stands for the dimension of $v_{i}\left(1\leq i\leq N-m+1\right).$

Secondly, define

(4) $$C_{i}^{m}=\frac{1}{N-m}\sum_{j=1,j\neq i}^{N-m+1}\Theta (r-∥v_{i}-v_{j}∥).$$

*r* stands for a tolerance value which is defined as $r=\varepsilon \cdot \sigma_{x}$, where ε is a small parameter and $\sigma_{x}$ is the standard deviation of x. Θ(·), the Heaviside function, which is defined as

(5) $$\Theta \left(x\right)=\left\{\begin{array}{cc} 0, & x<0;\\ 1, & x\geq 0. \end{array}\right.$$

∥·∥ represents the Chebyshev distance, i.e.,

(6) $$∥v_{i}-v_{j}∥=\max(|x_{i}-x_{j}\left|,\right|x_{i+1}-x_{j+1}\left|,\ldots ,\right|x_{i+m-1}-x_{j+m-1}\left|\right).$$

Similarly, define

(7) $$C_{i}^{m+1}=\frac{1}{N-m-1}\sum_{j=1,j\neq i}^{N-m}\Theta (r-∥v_{i}-v_{j}∥).$$

Thirdly, in view of Equations (4) and (7), we define

(8) $$U^{m}=\frac{1}{N-m+1}\sum_{i=1}^{N-m+1}C_{i}^{m},$$

and

(9) $$U^{m+1}=\frac{1}{N-m}\sum_{i=1}^{N-m}C_{i}^{m+1}.$$

Finally, calculate SampEn of x as

(10) $$\mathrm{SampEn}=-\ln\frac{U^{m+1}}{U^{m}}.$$

The value of SampEn is not less than 0, and a larger value of SampEn means more complexity [47]. Similar to our previous study [24,43], *m* and ε are fixed at 2 and 0.2, respectively.

In addition, because $d_{i,i,k}=1\left(i=1,2,\ldots ,160,k=1,2,\ldots ,923\right),$ the SampEn of $d_{i,i}$ equals 0 (i=1,2,…,160). Thus, for each participant, a SampEn matrix of size 160×160 is obtained (Figure 2). The SampEn matrix is labeled as

(11) $$E=\left[\begin{array}{ccc} e_{1,1} & \cdots & e_{1,160}\\ \vdots & \ddots & \vdots \\ e_{160,1} & \cdots & e_{160,160} \end{array}\right]=\left[\begin{array}{c} E_{1}\\ \vdots \\ E_{160} \end{array}\right].$$

The element $e_{i,j}$ represents the SampEn of dynamic FC between the *i*th ROI and *j*th ROI (i,j=1,2,…,160). $e_{i,i}$ equals 0 (i=1,2,…,160). The matrix *E* is used to cluster the 160 ROIs into 6 clusters.

### 2.6. Clustering ROIs into 6 Clusters by Applying the K-Means Clustering Algorithm to the Static FC Matrix

For each of the 31 participants, there exists a static FC matrix *B* of size 160×160. The *i*th (1≤i≤160) row $B_{i}=\left(b_{i,1},b_{i,2},\ldots ,b_{i,160}\right)$ represents the static FC between the *i*th ROI and all the other ROIs.

The K-means clustering algorithm is commonly used to cluster different observations into different clusters based on the distance between these observations [29]. In the present paper, the K-means clustering algorithm is applied to the matrix *B* to cluster 160 ROIs into 6 clusters. Procedures of the algorithm are briefly described as follows.

First, select 6 rows from the matrix *B* and use these 6 rows as initial cluster centroids.

Secondly, calculate the squared Euclidean distance between each row and each initial cluster centroid, and then assign each row to the cluster with the closest centroid.

Thirdly, when all rows have been assigned, calculate the average of the rows in each cluster to obtain 6 new cluster centroids.

Finally, repeat the second and the third steps until the centroids no longer change.

The algorithm generates 6 clusters, and each cluster is composed of different rows of the matrix *B* (or of different ROIs). Based on the 6 clusters, an individual adjacent matrix of size 160×160 is generated [36,41,42]. The individual adjacent matrix is labeled as

(12) $$F=\left[\begin{array}{ccc} f_{1,1} & \cdots & f_{1,160}\\ \vdots & \ddots & \vdots \\ f_{160,1} & \cdots & f_{160,160} \end{array}\right].$$

Each of the elements on the main diagonal of *F* is 1, and other elements of *F* are defined as follows: $f_{i,j}=1$ if the *i*th ROI and the *j*th ROI are contained in the same cluster and $f_{i,j}=0$ otherwise.

Since the study includes 31 participants, 31 individual adjacent matrices are obtained. A group adjacent matrix of size 160×160 is obtained by averaging 31 individual adjacent matrices. The group adjacent matrix is labeled as

(13) $$G=\left[\begin{array}{ccc} g_{1,1} & \cdots & g_{1,160}\\ \vdots & \ddots & \vdots \\ g_{160,1} & \cdots & g_{160,160} \end{array}\right].$$

The K-means clustering algorithm is further applied to the matrix *G* to obtain the group cluster result [36,41,42] and the 6 clusters of the group cluster result are compared with the 6 functional networks shown in Figure 1a.

The detailed clustering procedure is performed by MATLAB software (MATLAB R2014b). Considering that the K-means clustering algorithm is sensitive to the initial cluster centroids, we repeat each clustering procedure 500 times, and the cluster result with the lowest within-cluster distance is adopted.

### 2.7. Clustering ROIs into 6 Clusters by Applying the K-Means Clustering Algorithm to the SampEn Matrix

The procedures described in Section 2.6 are also applied to the SampEn matrix *E*, and 6 clusters are obtained.

## 3. Results

### 3.1. Six Clusters of ROIs for the Static FC

**Figure 3:**
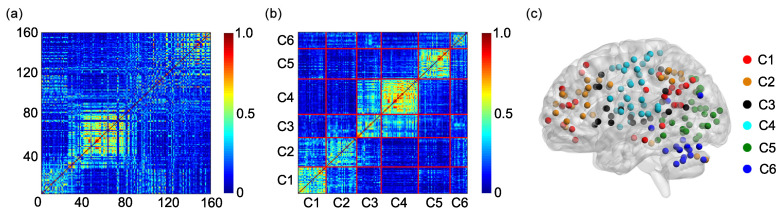
(**a**) The group adjacent matrix for the static FC. (**b**) The reorganization of the group adjacent matrix based on the 6 clusters obtained by applying the K-means clustering algorithm to the group adjacent matrix. Since the *i*th row and the *i*th column of the group adjacent matrix are reorganized simultaneously, the reorganized matrix is also symmetric. (**c**) The 6 clusters are shown on a surface rendering of the brain. C1: cluster 1; C2: cluster 2; C3: cluster 3; C4: cluster 4; C5: cluster 5; C6: cluster 6.

The group adjacent matrix for the static FC is shown in Figure 3a. The horizontal and vertical coordinates represent the sequential numbers of the ROIs. The sequential number and the name of each ROI can be found in Table A1 in Appendix A.

Rows of the group adjacent matrix can be clustered into six clusters by the K-means clustering algorithm (Figure 3b). The numbers of rows in clusters 1–6 are 26, 29, 23, 35, 30, and 17, respectively (Table 1). The ROIs in clusters 1–6 can be found in the third and fourth columns of Table A2, Table A3, Table A4, Table A5, Table A6 and Table A7 in Appendix A. Since each row of the adjacent matrix corresponds to a ROI, the six clusters can also be shown on a surface rendering of the brain (Figure 3c), which resembles Figure 1a to a large extent.

**Figure 4:**
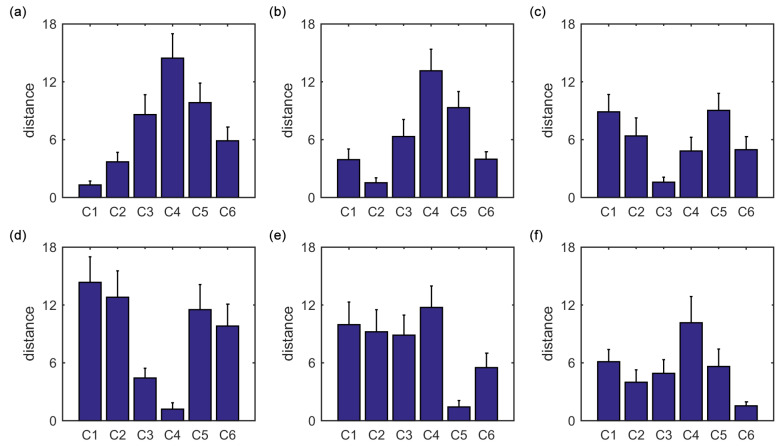
The average of the squared Euclidean distances from all ROIs in each of the six clusters to the centroid of cluster i(i=1,2,3,4,5,6). (**a**) Centroid of cluster 1. (**b**) Centroid of cluster 2. (**c**) Centroid of cluster 3. (**d**) Centroid of cluster 4. (**e**) Centroid of cluster 5. (**f**) Centroid of cluster 6. The error bars represent standard deviations.

The average of the squared Euclidean distances from all ROIs in each of the six clusters to the centroid of cluster i(i=1,2,3,4,5,6) is also evaluated, as shown in Figure 4a–f. For each centroid, among the six averaged distances, the averaged distance from the cluster i(i=1,2,3,4,5,6) to the centroid of cluster *i* is the lowest. This is consistent with the main idea of the K-means clustering algorithm.

### 3.2. The Overlap Ratios between the Six Clusters for the Static FC and the Six Functional Networks

The overlap ratios between each cluster and each functional network is analyzed in Table 1. The overlap ratios between cluster 1 and the default network, the frontal-parietal network, the cingulo-opercular network, the sensorimotor network, the occipital network, as well as the cerebellum network are 25/26 (≈96.15%), 0, 1/26 (≈3.85%), 0, 0, and 0, respectively. Obviously, the overlap ratio between cluster 1 and the default network is the highest. Thus, cluster 1 corresponds to the default network. Similarly, we can obtain that clusters 2–6, respectively, correspond to the frontal-parietal network, the cingulo-opercular network, the sensorimotor network, the occipital network, and the cerebellum network, with the overlap ratios, respectively, equaling 20/29 (≈68.97%), 21/23 (≈91.30%), 32/35 (≈91.43%), 22/30 (≈73.33%), and 14/17 (≈82.35%). These overlap ratios are high.

### 3.3. The Consistency Ratios between the Six Clusters for the Static FC and the Functional Networks

Based on the data shown in Table 1, the consistency between the cluster results and the functional networks can also be evaluated. The consistency ratio between cluster 1 and the default network is 25/(25 + 9 + 1) (≈71.43%), in which 9 is the number of ROIs in the default network but not in cluster 1, and 1 is the number of ROIs in cluster 1 but not in the default network. Similarly, we can obtain that the consistency ratios between cluster 2 and the frontal-parietal network, cluster 3 and the cingulo-opercular network, cluster 4 and the sensorimotor network, cluster 5 and the occipital network, and cluster 6 and the cerebellum network are 20/(20 + 1 + 9) (≈66.67%), 21/(21 + 11 + 2) (≈61.76%), 32/(32 + 1 + 3) (≈88.89%), 22/(22 + 0 + 8) (≈73.33%), and 14/(14 + 4 + 3) (≈66.67%), respectively. These consistency ratios are high.

### 3.4. Six Clusters of ROIs for the SampEn of Dynamic FC

**Figure 5:**
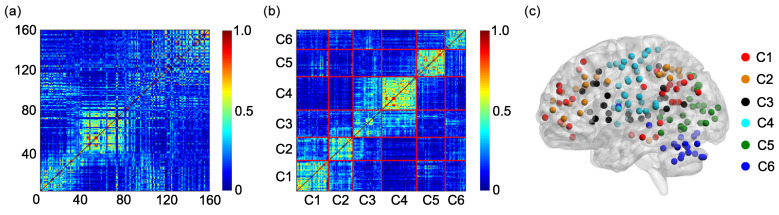
(**a**) The group adjacent matrix for the SampEn of dynamic FC. (**b**) The reorganization of the group adjacent matrix based on the six clusters obtained by applying the K-means clustering algorithm to the group adjacent matrix. Since the *i*th row and the *i*th column of the group adjacent matrix are reorganized simultaneously, the reorganized matrix is also symmetric. (**c**) The six clusters are shown on a surface rendering of the brain. C1: cluster 1; C2: cluster 2; C3: cluster 3; C4: cluster 4; C5: cluster 5; C6: cluster 6.

The group adjacent matrix for the SampEn of dynamic FC is presented in Figure 5a. The horizontal and vertical coordinates stand for the sequential numbers of the ROIs. The sequential number and the name of each ROI can be found in Table A1 in Appendix A.

Rows of the group adjacent matrix can be divided into six clusters by the K-means clustering algorithm (Figure 5b). The numbers of rows in clusters 1–6 are 30, 23, 27, 33, 27, and 20, respectively (Table 2). The ROIs in clusters 1–6 can be found in the fifth and sixth columns of Table A2, Table A3, Table A4, Table A5, Table A6 and Table A7 in Appendix A. The six clusters can also be shown on a surface rendering of the brain (Figure 5c), which resembles Figure 1a and Figure 3c to a large extent.

**Figure 6:**
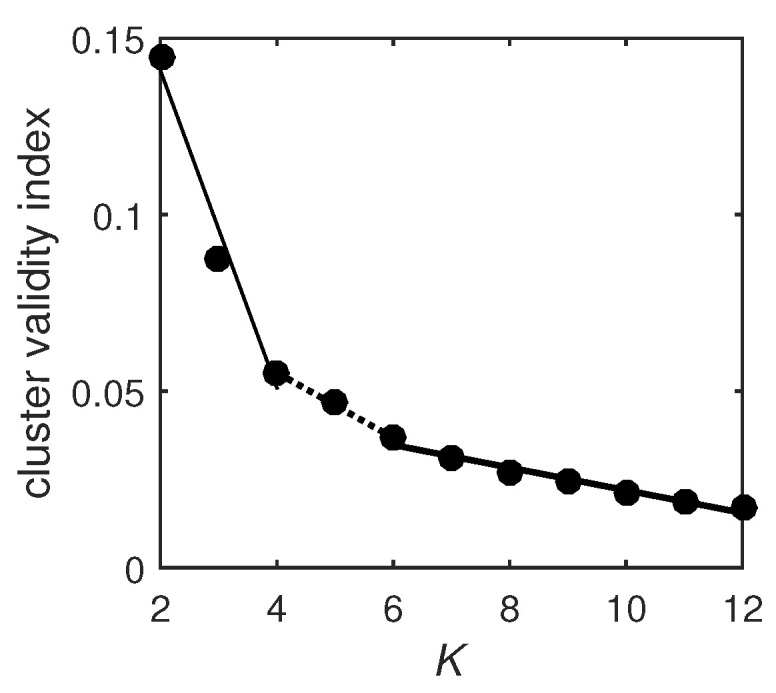
The dependence of the cluster validity index on *K*. The thin solid, dotted, and bold solid lines are trend lines of the filled circles. Since slopes of the trend lines change significantly at K= 4 and 6, based on the elbow criterion, the optimal values of *K* are 4 and 6.

Furthermore, other values of K(K=2,…,12) are also tried in the K-means clustering algorithm, and the optimal value of *K* is determined by the elbow criterion of the cluster validity index, which is defined as the ratio of within-cluster distances to between-cluster distances [15,20,27]. The dependence of the cluster validity index on *K* is shown in Figure 6. It is seen that two elbows appear at K= 4 and 6 due to the changes of slopes of the trend lines. Thus, the optimal values of *K* are 4 and 6. In order to compare the cluster results with the six functional networks already discussed in the literature [25], *K* is fixed at 6 in the present paper.

**Figure 7:**
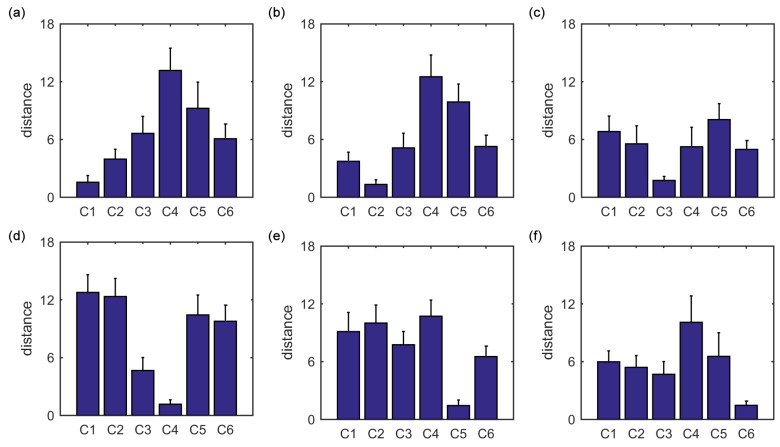
The average of the squared Euclidean distances from all ROIs in each of the six clusters to the centroid of cluster i(i=1,2,3,4,5,6). (**a**) Centroid of cluster 1. (**b**) Centroid of cluster 2. (**c**) Centroid of cluster 3. (**d**) Centroid of cluster 4. (**e**) Centroid of cluster 5. (**f**) Centroid of cluster 6. The error bars represent standard deviations.

The average of the squared Euclidean distances from all ROIs in each of the six clusters to the centroid of cluster i(i=1,2,3,4,5,6) is calculated, as shown in Figure 7a–f. For each centroid, among the six averaged distances, the averaged distance from the cluster i(i=1,2,3,4,5,6) to the centroid of cluster *i* is the lowest. This is also in line with the main idea of the K-means clustering algorithm.

### 3.5. The Overlap Ratios between the Six Clusters for the SampEn of Dynamic FC and the Six Functional Networks

The overlap ratio between each cluster and each functional network is analyzed in Table 2. By evaluating the overlap ratio between each cluster and each functional network, we find that clusters 1–6, respectively, correspond to the default network, the frontal-parietal network, the cingulo-opercular network, the sensorimotor network, the occipital network, and the cerebellum network, with the overlap ratios, respectively, equaling 29/30 (≈96.67%), 20/23 (≈86.96%), 23/27 (≈85.19%), 30/33 (≈90.91%), 22/27 (≈81.48%), and 18/20 (≈90.00%). These overlap ratios are very high.

### 3.6. The Consistency Ratios between the Six Clusters for the SampEn of Dynamic FC and the Six Functional Networks

Based on the data shown in Table 2, the consistency ratios between the six clusters obtained from the SampEn of dynamic FC and the six functional networks are evaluated. The consistency ratios between cluster 1 and the default network, cluster 2 and the frontal-parietal network, cluster 3 and the cingulo-opercular network, cluster 4 and the sensorimotor network, cluster 5 and the occipital network, and cluster 6 and the cerebellum network are 29/(29 + 5 + 1) (≈82.86%), 20/(20 + 1 + 3) (≈83.33%), 23/(23 + 9 + 4) (≈63.89%), 30/(30 + 3 + 3) (≈83.33%), 22/(22 + 0 + 5) (≈81.48%), and 18/(18 + 0 + 2) (≈90.00%), respectively. These consistency ratios are very high.

### 3.7. The SampEn of Dynamic FC is More Effective Than the Static FC

**Figure 8:**
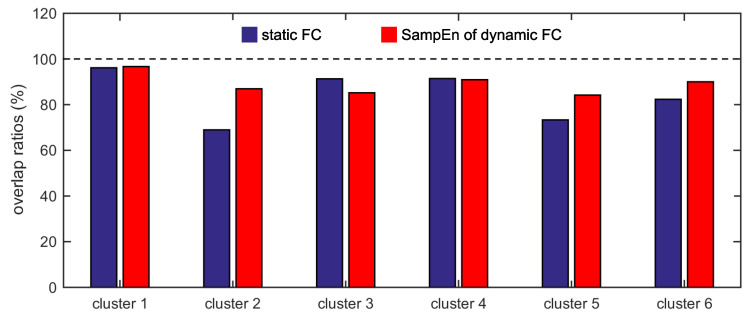
The overlap ratios between cluster 1 and the default network, cluster 2 and the frontal-parietal network, cluster 3 and the cingulo-opercular network, cluster 4 and the sensorimotor network, cluster 5 and the occipital network, and cluster 6 and the cerebellum network for the two different measurements.

For the two different measurements (the static FC and the SampEn of dynamic FC), the overlap ratios between cluster 1 and the default network, cluster 2 and the frontal-parietal network, cluster 3 and the cingulo-opercular network, cluster 4 and the sensorimotor network, cluster 5 and the occipital network, and cluster 6 and the cerebellum network are shown in Figure 8. For cluster 3, the overlap ratio corresponding to the static FC (91.30%) is larger than that corresponding to the SampEn of dynamic FC (85.19%). For cluster 4, the overlap ratio corresponding to the static FC (91.43%) is slightly larger than that corresponding to the SampEn of dynamic FC (90.91%). For the other four clusters (clusters 1, 2, 5, and 6), the overlap ratios corresponding to the SampEn of dynamic FC are larger than that corresponding to the static FC. For clusters 1, 2, 5, and 6, the overlap ratios corresponding to the SampEn of dynamic FC are 96.67%, 86.96%, 81.48%, and 90.00%, whereas the overlap ratios corresponding to the static FC are 96.15%, 68.97%, 73.33%, and 82.35%.

**Figure 9:**
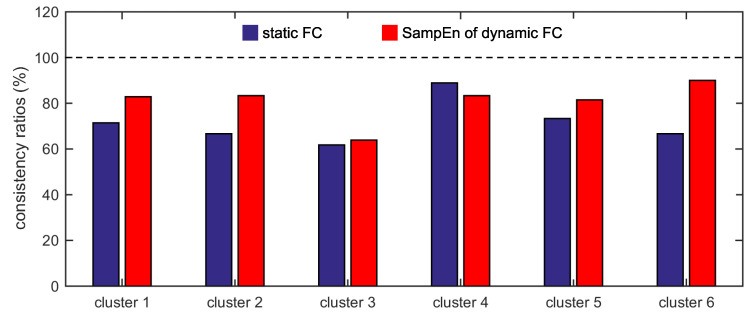
The consistency ratios between cluster 1 and the default network, cluster 2 and the frontal-parietal network, cluster 3 and the cingulo-opercular network, cluster 4 and the sensorimotor network, cluster 5 and the occipital network, and cluster 6 and the cerebellum network for the two different measurements.

For the two different measurements, the consistency ratios between cluster 1 and the default network, cluster 2 and the frontal-parietal network, cluster 3 and the cingulo-opercular network, cluster 4 and the sensorimotor network, cluster 5 and the occipital network, and cluster 6 and the cerebellum network are shown in Figure 9. For cluster 4, the consistency ratio corresponding to the static FC (88.89%) is larger than that corresponding to the SampEn of dynamic FC (83.33%). For the other five clusters, the consistency ratios corresponding to the SampEn of dynamic FC are larger than that corresponding to the static FC. For clusters 1, 2, 3, 5, and 6, the consistency ratios corresponding to the SampEn of dynamic FC are 82.86%, 83.33%, 63.89%, 81.48%, and 90.00%, whereas the consistency ratios corresponding to the static FC are 71.43%, 66.67%, 61.76%, 73.33%, and 66.67%.

According to the results shown in Figure 8 and Figure 9, we conclude that the SampEn of dynamic FC is more effective than the static FC in clustering different ROIs into different functional networks. This phenomenon can be interpreted by evaluating the similarity between the adjacent matrix generated based on the six functional networks (Figure 1b) and the group adjacent matrix for the static FC (Figure 3a) or for the SampEn of dynamic FC (Figure 5a). The similarity is evaluated by the squared Euclidean distance, and a smaller distance means more similarity. The distances from the adjacent matrix shown in Figure 1b to the group adjacent matrices shown in Figure 3a and in Figure 5a are 2409.58 and 2376.52, respectively. The latter is smaller than the former, i.e., the similarity between the adjacent matrix shown in Figure 1b and the group adjacent matrix shown in Figure 5a is larger than the similarity between the adjacent matrix shown in Figure 1b and the group adjacent matrix shown in Figure 3a. This causes the SampEn of dynamic FC to be more effective than the static FC in clustering different ROIs into different functional networks.

## 4. Conclusions and Discussion

Different brain regions in the human brain functionally interact with each other to construct multiple functional networks. Identifying the function of each functional network and the brain regions contained in each functional network is very important for understanding the human brain. The present study tests the feasibility of using the K-means clustering algorithm to identify the functional networks based on the FC, including the static FC and the dynamic FC. By applying the K-means clustering algorithm to the static FC or the SampEn of dynamic FC between different ROIs determined by the Dosenbach’s template, we show that the Dosenbach’s 160 ROIs can be divided into six clusters which show high overlap and consistency ratios with the six functional networks identified by applying modularity optimization on the average FC matrix across a large cohort of healthy subjects. The results indicate that the combination of the K-means clustering algorithm and the FC can identify the functional networks of the human brain. The K-means algorithm has been commonly used to parcellate cortical or subcortical regions based on the static FC [30,31,32,33,34,35,36,37,38]. These previous studies along with the present study extend the application of machine learning methods in brain sciences.

Furthermore, we show that, for four of six clusters, the overlap ratios corresponding to the SampEn of dynamic FC are larger than that corresponding to the static FC, and for five of six clusters, the consistency ratios corresponding to the SampEn of dynamic FC are larger than that corresponding to the static FC. This indicates that nonlinear dynamic characteristics of the FC is more effective than the static characteristics of the FC in identifying brain functional networks. In our previous studies, by characterizing the nonlinear characteristics of dynamic FC in healthy subjects and patients with schizophrenia, we have shown that SampEn of the amygdala-cortical FC in healthy subjects decreased with age increasing, and the visual cortex of the patients with schizophrenia exhibited significantly higher SampEn than that of the healthy subjects [24,43]. In the future, nonlinear characteristics of dynamic FC should be deeply used to characterize properties of brain functional networks and the complexity of the human brain.

## Figures and Tables

**Table 1:** The number of ROIs in the overlapping part between each functional network and each cluster obtained from the static FC.

	Cluster 1 (n=26)	Cluster 2 (n=29)	Cluster 3 (n=23)	Cluster 4 (n=35)	Cluster 5 (n=30)	Cluster 6 (n=17)
Default (n=34)	25	2	0	0	6	1
Frontal-Parietal (n=21)	0	20	1	0	0	0
Cingulo-Percular (n=32)	1	5	21	3	0	2
Sensorimotor (n=33)	0	0	1	32	0	0
Occipital (n=22)	0	0	0	0	22	0
Cerebellum (n=18)	0	2	0	0	2	14

**Table 2:** The number of ROIs in the overlapping part between each functional network and each cluster obtained from the SampEn of dynamic FC.

	Cluster 1 (n=30)	Cluster 2 (n=23)	Cluster 3 (n=27)	Cluster 4 (n=33)	Cluster 5 (n=27)	Cluster 6 (n=20)
Default (n=34)	29	0	0	0	5	0
Frontal-parietal (n=21)	0	20	1	0	0	0
Cingulo-percular (n=32)	1	3	23	3	0	2
Sensorimotor (n=33)	0	0	3	30	0	0
Occipital (n=22)	0	0	0	0	22	0
Cerebellum (n=18)	0	0	0	0	0	18

## Appendix A

**Table A1:** The names and the sequential numbers of 160 ROIs.

No.	Name	No.	Name	No.	Name	No.	Name
1	vmPFC	41	pre-SMA	81	fusiform	121	inf cerebellum
2	aPFC	42	vFC	82	temporal	122	inf cerebellum
3	aPFC	43	SMA	83	temporal	123	temporal
4	mPFC	44	mid insula	84	fusiform	124	angular gyrus
5	aPFC	45	frontal	85	precuneus	125	TPJ
6	vmPFC	46	precentral gyrus	86	sup parietal	126	occipital
7	vmPFC	47	thalamus	87	precuneus	127	med cerebellum
8	aPFC	48	mid insula	88	IPL	128	lat cerebellum
9	vent aPFC	49	precentral gyrus	89	parietal	129	occipital
10	vent aPFC	50	parietal	90	post cingulate	130	med cerebellum
11	vmPFC	51	precentral gyrus	91	inf temporal	131	inf cerebellum
12	vlPFC	52	precentral gyrus	92	occipital	132	precuneus
13	vmPFC	53	precentral gyrus	93	post cingulate	133	occipital
14	ACC	54	parietal	94	precuneus	134	IPS
15	vlPFC	55	mid insula	95	temporal	135	occipital
16	dlPFC	56	mid insula	96	IPL	136	occipital
17	sup frontal	57	thalamus	97	parietal	137	occipital
18	vPFC	58	thalamus	98	lat cerebellum	138	med cerebellum
19	ACC	59	mid insula	99	post parietal	139	occipital
20	sup frontal	60	temporal	100	sup temporal	140	inf cerebellum
21	ACC	61	mid insula	101	IPL	141	occipital
22	dlPFC	62	parietal	102	angular gyrus	142	occipital
23	vPFC	63	inf temporal	103	temporal	143	med cerebellum
24	dlPFC	64	parietal	104	IPL	144	med cerebellum
25	vFC	65	parietal	105	precuneus	145	occipital
26	ant insula	66	parietal	106	occipital	146	occipital
27	dACC	67	precentral gurus	107	IPL	147	occipital
28	ant insula	68	temporal	108	post cingulate	148	occipital
29	dFC	69	parietal	109	lat cerebellum	149	occipital
30	basal ganglia	70	post insula	110	inf cerebellum	150	inf cerebellum
31	mFC	71	basal ganglia	111	post cerebellum	151	inf cerebellum
32	frontal	72	inf temporal	112	precuneus	152	post occipital
33	vFC	73	post cingulate	113	lat cerebellum	153	post occipital
34	dFC	74	parietal	114	IPS	154	post occipital
35	dFC	75	parietal	115	post cingulate	155	inf cerebellum
36	dFC	76	post insula	116	IPS	156	post occipital
37	vFC	77	parietal	117	angular gyrus	157	post occipital
38	basal ganglia	78	temporal	118	occipital	158	post occipital
39	basal ganglia	79	post parietal	119	occipital	159	post occipital
40	vFC	80	post cingulate	120	med cerebellum	160	post occipital

**Table A2:** ROIs in the default network and in cluster 1 for the static FC and the SampEn of dynamic FC. ROIs in cluster 1 but not in the default network are marked by underlines.

Default Network (n=34)		Cluster 1 for the Static FC (n=26)		Cluster 1 for the SampEn (n=30)	
ROI 1	ROI 92	ROI 1		ROI 1	
ROI 4	ROI 93	ROI 4	ROI 93	ROI 4	ROI 93
ROI 5	ROI 94		ROI 94	ROI 5	ROI 94
ROI 6	ROI 105	ROI 6	ROI 105	ROI 6	ROI 105
ROI 7	ROI 108	ROI 7	ROI 108	ROI 7	ROI 108
ROI 11	ROI 111	ROI 11	ROI 111	ROI 11	ROI 111
ROI 13	ROI 112	ROI 13	ROI 112	ROI 13	ROI 112
ROI 14	ROI 115	ROI 14	ROI 115	ROI 14	ROI 115
ROI 15	ROI 117	ROI 15	ROI 117	ROI 15	ROI 117
ROI 17	ROI 124	ROI 17	ROI 124	ROI 17	ROI 124
ROI 20	ROI 132	ROI 20		ROI 20	
ROI 63	ROI 134	ROI 63	ROI 134	ROI 63	ROI 134
ROI 72	ROI 136	ROI 72		ROI 72	
ROI 73	ROI 137	ROI 73		ROI 73	ROI 137
ROI 84	ROI 141			ROI 84	
ROI 85	ROI 146	ROI 85		ROI 85	ROI 146
ROI 90			ROI 102		ROI 102
ROI 91		ROI 91		ROI 91	

**Table A3:** ROIs in the frontal-parietal network and in cluster 2 for the static FC and the SampEn of dynamic FC. ROIs in cluster 2 but not in the frontal-parietal network are marked by underlines.

Frontal-Parietal Network (n=21)		Cluster 2 for the Static FC (n=29)		Cluster 2 for the SampEn (n=23)	
ROI 2	ROI 99	ROI 2	ROI 99	ROI 2	ROI 99
ROI 3	ROI 101	ROI 3	ROI 101	ROI 3	ROI 101
ROI 9	ROI 104	ROI 9	ROI 104	ROI 9	ROI 104
ROI 10	ROI 107	ROI 10	ROI 107	ROI 10	ROI 107
ROI 12	ROI 114	ROI 12	ROI 114	ROI 12	ROI 114
ROI 16	ROI 116	ROI 16	ROI 116	ROI 16	ROI 116
ROI 21		ROI 21	ROI 5	ROI 21	ROI 8
ROI 22		ROI 22	ROI 8	ROI 22	ROI 18
ROI 23		ROI 23	ROI 18	ROI 23	ROI 81
ROI 24		ROI 24	ROI 19	ROI 24	
ROI 29		ROI 29	ROI 25	ROI 29	
ROI 34			ROI 81		
ROI 36		ROI 36	ROI 137	ROI 36	
ROI 88		ROI 88	ROI 140	ROI 88	
ROI 96		ROI 96	ROI 155	ROI 96	

**Table A4:** ROIs in the cingulo-percular network and in cluster 3 for the static FC and the SampEn of dynamic FC. ROIs in cluster 3 but not in the cingulo-percular network are marked by underlines.

Cingulo-Percular Network (n=32)		Cluster 3 for the Static FC (n=23)		Cluster 3 for the SampEn (n=27)	
ROI 8	ROI 61		ROI 61		ROI 61
ROI 18	ROI 71		ROI 71		ROI 71
ROI 19	ROI 76			ROI 19	
ROI 25	ROI 78		ROI 78	ROI 25	ROI 78
ROI 26	ROI 80	ROI 26		ROI 26	
ROI 27	ROI 81	ROI 27		ROI 27	
ROI 28	ROI 87	ROI 28	ROI 87	ROI 28	ROI 87
ROI 30	ROI 89	ROI 30	ROI 89	ROI 30	ROI 89
ROI 31	ROI 95	ROI 31	ROI 95	ROI 31	ROI 95
ROI 33	ROI 97	ROI 33	ROI 97	ROI 33	ROI 97
ROI 38	ROI 100	ROI 38		ROI 38	ROI 100
ROI 39	ROI 102	ROI 39		ROI 39	
ROI 40	ROI 103	ROI 40	ROI 103	ROI 40	ROI 103
ROI 44	ROI 125		ROI 125		ROI 125
ROI 47		ROI 47	ROI 32		ROI 32
ROI 57		ROI 57	ROI 34	ROI 57	ROI 34
ROI 58		ROI 58		ROI 58	ROI 35
ROI 59					ROI 37

**Table A5:** ROIs in the sensorimotor network and in cluster 4 for the static FC and the SampEn of dynamic FC. ROIs in cluster 4 but not in the sensorimotor network are marked by underlines.

Sensorimotor Network (n=33)		Cluster 4 for the Static FC (n=35)		Cluster 4 for the SampEn (n=33)	
ROI 32	ROI 62		ROI 62		ROI 62
ROI 35	ROI 64	ROI 35	ROI 64		ROI 64
ROI 37	ROI 65	ROI 37	ROI 65		ROI 65
ROI 41	ROI 66	ROI 41	ROI 66	ROI 41	ROI 66
ROI 42	ROI 67	ROI 42	ROI 67	ROI 42	ROI 67
ROI 43	ROI 68	ROI 43	ROI 68	ROI 43	ROI 68
ROI 45	ROI 69	ROI 45	ROI 69	ROI 45	ROI 69
ROI 46	ROI 70	ROI 46	ROI 70	ROI 46	ROI 70
ROI 48	ROI 74	ROI 48	ROI 74	ROI 48	ROI 74
ROI 49	ROI 75	ROI 49	ROI 75	ROI 49	ROI 75
ROI 50	ROI 77	ROI 50	ROI 77	ROI 50	ROI 77
ROI 51	ROI 79	ROI 51	ROI 79	ROI 51	ROI 79
ROI 52	ROI 82	ROI 52	ROI 82	ROI 52	ROI 82
ROI 53	ROI 83	ROI 53	ROI 83	ROI 53	ROI 83
ROI 54	ROI 86	ROI 54	ROI 86	ROI 54	ROI 86
ROI 55		ROI 55	ROI 44	ROI 55	ROI 44
ROI 56		ROI 56	ROI 59	ROI 56	ROI 59
ROI 60		ROI 60	ROI 76	ROI 60	ROI 76

**Table A6:** ROIs in the occipital network and in cluster 5 for the static FC and the SampEn of dynamic FC. ROIs in cluster 5 but not in the occipital network are marked by underlines.

Occipital Network (n=22)		Cluster 5 for the Static FC (n=30)		Cluster 5 for the SampEn (n=27)	
ROI 106	ROI 153	ROI 106	ROI 153	ROI 106	ROI 153
ROI 118	ROI 154	ROI 118	ROI 154	ROI 118	ROI 154
ROI 119	ROI 156	ROI 119	ROI 156	ROI 119	ROI 156
ROI 123	ROI 157	ROI 123	ROI 157	ROI 123	ROI 157
ROI 126	ROI 158	ROI 126	ROI 158	ROI 126	ROI 158
ROI 129	ROI 159	ROI 129	ROI 159	ROI 129	ROI 159
ROI 133	ROI 160	ROI 133	ROI 160	ROI 133	ROI 160
ROI 135		ROI 135	ROI 84	ROI 135	ROI 90
ROI 139		ROI 139	ROI 90	ROI 139	ROI 92
ROI 142		ROI 142	ROI 92	ROI 142	ROI 132
ROI 145		ROI 145	ROI 132	ROI 145	ROI 136
ROI 147		ROI 147	ROI 136	ROI 147	ROI 141
ROI 148		ROI 148	ROI 138	ROI 148	
ROI 149		ROI 149	ROI 141	ROI 149	
ROI 152		ROI 152	ROI 143	ROI 152	

**Table A7:** ROIs in the cerebellum network and in cluster 6 for the static FC and the SampEn of dynamic FC. ROIs in cluster 6 but not in the cerebellum network are marked by underlines.

Cerebellum Network (n=18)		Cluster 6 for the Static FC (n=17)		Cluster 6 for the SampEn (n=20)	
ROI 98	ROI 138	ROI 98		ROI 98	ROI 138
ROI 109	ROI 140	ROI 109		ROI 109	ROI 140
ROI 110	ROI 143	ROI 110		ROI 110	ROI 143
ROI 113	ROI 144	ROI 113	ROI 144	ROI 113	ROI 144
ROI 120	ROI 150	ROI 120	ROI 150	ROI 120	ROI 150
ROI 121	ROI 151	ROI 121	ROI 151	ROI 121	ROI 151
ROI 122	ROI 155	ROI 122		ROI 122	ROI 155
ROI 127		ROI 127	ROI 80	ROI 127	ROI 47
ROI 128		ROI 128	ROI 100	ROI 128	ROI 80
ROI 130		ROI 130	ROI 146	ROI 130	
ROI 131		ROI 131		ROI 131	
